# Intergenerational differences in the urban vibrancy of TOD: Impacts of the built environment on the activities of different age groups

**DOI:** 10.3389/fpubh.2022.994835

**Published:** 2022-09-06

**Authors:** Bingjie Yu, Xu Cui, Runze Liu, Pinyang Luo, Fangzhou Tian, Tian Yang

**Affiliations:** School of Architecture, Southwest Jiaotong University, Chengdu, China

**Keywords:** mobile phone signaling data, intergenerational differences, urban vibrancy, TOD, metro, built environment

## Abstract

Transit-oriented development (TOD) has been regarded as an effective way to improve urban vibrancy and facilitate affordable, equitable, and livable communities in metro station areas (MSAs). Previous studies placed great attention on the interplay between the MSA-level built environment and overall human activities while neglecting the heterogeneity among different age groups. To address this gap, we leverage the mobile phone signaling data to quantify the spatio-temporal distribution of the MSA-level human activities among different age groups as measured by the vibrancy index (VI). Furthermore, we investigate the impact of the MSA-level built environment on the VI and its intergenerational differences by employing multiple linear regressions based on multi-sourced data. To this end, Chengdu—a TOD-thriving megacity in China—is chosen as a case study. The results indicate that: (1) Residential and bus stop density are positively associated with the VI. And the magnitudes of the correlation coefficients are similar among different age groups. (2) Distance to CBD is negatively associated with the VI of teenagers (12–18 years), middle-aged adults (40–59 years), and older adults (above 60 years) but unrelated to the VI of young adults (19–39 years). (3) Employment density is positively associated with the VI of young and middle-aged adults but insignificantly associated with the VI of teenagers and older adults. (4) The correlations between the floor area ratio and the VI are positive for all age groups. As age increases, the significance of such correlations becomes more pronounced. (5) Streetscape greenery shows a more significant positive correlation with the VI of teenagers and older adults as compared to those of young and middle-aged adults. (6) Significant negative correlations exist between housing price and the VI of different age groups. The findings can inform the development and design of vibrant TOD communities.

## Introduction

In today's society, where urban life is highly socialized, the value of space is determined by its usage frequency, i.e., urban vibrancy (1). The concept of urban vibrancy was first introduced by Jacobs in her seminal text - *The Death and Life of Great American Cities* (2). She regarded urban vibrancy as the intensity and diversity of human activities in urban space. Moreover, she argued that the interaction processes of activities and living places form the diversity of cities. Urban vibrancy reflects the attractiveness, accessibility, and spatial quality of urban areas (3). It can facilitate social interactions (4), enhance cities' competitiveness, promote cities' economic growth and thus contribute to achieving sustainable urban development goals (5). Hence, how to stimulate urban vibrancy and improve the efficiency of space use in urban areas by leveraging built environment interventions is of great importance to the urban planning and construction process.

Under the green transport and new-type urbanization development strategies, urban rail transit is moving from the basic function of “meeting travel demand” toward the strategic function of “guiding urban development” (6). Given metro systems are the backbone of urban public transportation systems and attract millions of rail commuters every day, metro station areas (MSA) act as substantial transportation nodes for cities (7) and the impetus shaping the vibrancy of urban areas (8). The high density of passenger flows and businesses around metro stations generates considerable agglomeration and spillover effects, which in turn frame the MSA to be the hotspot area for urban activities and interactions (9). Therefore, research on the relationship between the built environment of MSA and urban vibrancy will have significant implications for informing the planning and design process of transit-oriented development (TOD).

Transit-oriented development concept aims to build vibrant communities and drive sustainable city development through high-capacity public transport, mixed land-use, and high-intensity construction (10). In recent years, the burgeoning development of information and communication technologies (ICT) has accelerated the emergence and prevalence of multi-sourced spatio-temporal data, which possesses great potential for measuring and quantifying urban vibrancy (11). Besides, the study of urban vibrancy has received wide scholarly attention from several disciplines, including urban planning (3), transportation (12), and urban geography (13). Also, the focus of integrated transport and land use planning has shifted from “providing amenities and facilities” toward “accommodating the needs of residents” (14). Against this background, MSA, as one of the most important carriers of urban vibrancy with intensive human activities, is attracting more and more attention among practitioners and scholars. For example, Chengdu - a TOD-informed megacity in China - has carried out initiatives for building 24-h vibrant communities in the MSA in its recently-released planning guidelines such as “Land Management Measures for Comprehensive Development of Chengdu Metro Rail Transit Stations” and “15-Minute Living Service Circle of TOD”. In addition, previous studies also show that the rational design of pedestrian systems (15) and appropriate layout of functional facilities and land use (12) can help enhance the intensity of human activities in the MSA.

It is worth noting that most of the existing studies use social media data (16), the heat map of mobile applications (17), economic activity data, and point-of-interest (POI) data to measure TOD vibrancy. However, these data fail to record the staying time and activity trajectories of individuals/groups due to their transient and cross-sectional features, leading to inaccurate identifications. Besides, previous studies extensively explore the impact of 5D built environment factors (i.e., density, diversity, design, distance to transit, and destination accessibility) (18, 19). However, it remains uncertain how refined built environment factors such as the streetscape and street network topology contribute to TOD vibrancy. Moreover, there are variation of different age groups in mobility. Although previous studies placed great attention on the interplay between the MSA-level built environment and overall human activities, the heterogeneity among different age groups has been neglected. This may result in the homogenization of the construction and residential population structure in the TOD.

To address these gaps, we set out to investigate the impact of the MSA-level built environment on the VI and its intergenerational differences using Chengdu as a case study based on multi-sourced data. To be specific, we first take advantage of the cellular signaling data to quantify the spatio-temporal distribution of the MSA-level human activities among different age groups as measured by the vibrancy index (VI). Then, we measure a wide range of refined built environment factors by leveraging various spatial data such as the POI data, street view data, and parcel-level land use data. Finally, we employ multiple linear regressions to model the relationship between the MSA-level built environment and the VI of different age groups. The empirical findings are expected to provide evidence-based guidance for the planning and design of TOD vibrant communities. The main contributions of this study are as follows: (1) quantifying the spatio-temporal distribution of the MSA-level VI among different age groups; (2) deciphering the impact of the MSA-level built environment on the VI and its intergenerational differences; (3) providing policy recommendations for accommodating affordable, livable, and equitable communities around metro stations.

The following of this paper is structured as follows: Section “Literature review” reviews and analyzes the literature on the urban vibrancy and its built environment determinants. Section “Study area and data” introduces the study area, data, and methodologies. Section “Results” presents the spatio-temporal distribution of the MSA-level human activities among different age groups and the results of regression analysis. Section “Discussion” interprets the results and carries out discussions. Section “Conclusions” concludes and provides evidence-based policy implications.

## Literature review

### Definition and measurement of urban vibrancy

Urban vibrancy is derived from Jacobs' (2) concept of street vibrancy. It means the intensity and type of people and their activities in a given space, which reflects the attractiveness and diversity of a city. Lynch (20) argued that urban vibrancy consists of three components: urban spatial form, functional composition, and social activities. Landry (21) considered that measures of urban vibrancy reflect the social-economic situation, physical environment, and interactions between society and the economy. Montgomery (22) provided a more comprehensive explanation of urban vibrancy. He treated urban vibrancy as a continuous and compact flow of people, efficient space use, and vibrant street cultural life. Moreover, Chhetri et al. (23) further suggested that urban vibrancy is an external manifestation of the interaction between residents and their surrounding space. From the above scholars' definitions of urban vibrancy, it can be seen that although the concept of urban vibrancy is under development, it is always based on human activities and interactions.

However, there are no uniform standards to measure or evaluate urban vibrancy. Some previous studies have used static data sources, including population censuses, interview surveys, housing prices, and Nighttime light (NTL) to characterize urban vibrancy (24–29), while these traditional data only reflect the static, rather than the dynamic, urban vibrancy. Besides, there are disadvantages of the traditional vibrancy characterization data, such as insufficient precision and small spatial coverage (30, 31). In recent years, with the rapid development of information and communication technology (ICT), multi-source big data provides the data basis for calculable and quantitative analysis of urban vibrancy (32). In this era of big data, mobile internet users are both the recipients and producers of data. The “digital geographic footprint” of cell phone users (33, 34) and social network information form a huge amount of big data on human spatio-temporal activities. Compared to traditional community census and survey data, big data has the advantages of timeliness, high penetration, large sample size, and abundant activity information (35). In this regard, many studies have used mobile phone signaling data (36), social media data (37), smartcard data (38), and POIs (34, 39) to explore the urban vibrancy. For instance, Chen et al. (1) used Facebook API to obtain the activity characteristics of residents in Hong Kong. They found that the north coast of Hong Kong Island and the south coast of Kowloon Peninsula were the most vibrant areas of the city. Yue et al. (40) used the diversity of POIs to measure the spatial vibrancy of the city. Guo et al. (41) analyzed the 24-h neighborhood vibrancy distribution in Xining, China, based on mobile base station communication records of 560,000 cell phone users. They found that the spatial and temporal street vibrancy is basically consistent with the rhythm of residents' daily life. Plus, other scholars aggregate a variety of data to reflect the comprehensive urban vibrancy (34). Xiao et al. (42) constructed a TOD vibrancy aggregation index using Sina Weibo data, Dianping data, subway smartcards, and Baidu heat maps. Huang et al. (35) measured pedestrian density, economic activity intensity, and social activity intensity in Shanghai through cell phone GPS, public service comments data, and social media data.

It is worth noting that the studies above all used multiple sources of big data to reflect urban vibrancy. However, these data sources are mostly transient, such as online check-in data (Sina Weibo). Data sources like this are limited in coverage and fail to reflect the staying time and activity trajectory of individuals/groups in MSAs due to being transient, which may lead to inaccurate identification of urban vibrancy. So large numbers of pass-through and short-time staying people flow being recorded as urban vibrancy. Meanwhile, these people do not consume, relax, live or work in MSAs, which cannot simply be defined as urban vibrancy. Secondly, little has been done to distinguish the heterogeneity of human activities by age in these studies. According to related research (43, 44), there are differences in the activity ability, activity scope, and activity purpose of different age groups. For example, the main activity spaces of teenagers are schools and home, the main activity spaces of youth are mainly home and employment places, while the main activity spaces of older adults are mostly home and community parks. Therefore, if we ignore the intergenerational differences in the impact of MSA-level built environment features on vibrancy, it may lead to overestimation/underestimation of the comprehensive influence of built environment elements on TOD vibrancy.

### Impact of the built environment on TOD vibrancy

The transport-oriented development policies are considered as the important impetus that enhances urban vibrancy (10). Related studies indicate that 5Ds built environment indicators affect the vibrancy of TOD (9, 12, 17, 39, 45). Scholars have conducted studies on the relationship between the built environment and TOD vibrancy in cities with developed rail transit, such as Shenzhen (9, 12, 42, 45), Hong Kong (15, 46), Shanghai (17), Singapore (9), and Montreal (47). For example, Ye et al. (17) explored the linkage mechanisms between the built environment of Shanghai's MSA and the Baidu heat map. They found that the vibrancy of TOD has a significant relationship with FAR, land-use mix, and bus station density. Zhou and Yang's (45) study pointed out that the service ability of TOD network influences the number of public facilities (POI density). In Xiao et al.'s (42) work, gradient boosting decision trees were used to explore the nonlinear synergistic effects of the built environment and TOD vibrancy in Shenzhen's MSAs. The study showed that the adequate transit service system, building density, and land-use mix were the most important drive factors of TOD vibrancy. They also found that different built environment indicators had synergistic promotion effects on TOD vibrancy. Similarly, a study by Yang et al. (12) revealed a non-linear trend in the impact of the metro station's proximity on urban vibrancy in Shenzhen. In the range of 0–810 m to the station, the station proximity negatively correlated with human activities, and there is no effect when beyond a threshold. The conclusion is consistent with the basic explanation of the ground rent theory curve. Moreover, Tu et al. (9) used multi-source big data such as smartcard data, Weibo check-in data, Twitter tweets, and EZ-link transaction tweet data as the characterization of the metro station vibrancy in three cities: Shenzhen, London, and Singapore. The study showed that the metro station vibrancy in central urban areas is higher than that in suburban areas and that cities with higher GDP have higher vibrancy. They also concluded that built environment factors such as road density, bus station density, land-use mix, and gross building area have a significant impact on the urban vibrancy aggregative indicators. Jacobs-Crisioni et al. (48) pointed out that built environment indicators, including metro station, railway station, and business density, have a positive effect on vibrancy intensity. In addition to the study of human activities at the ground level of metro stations, some scholars have conducted studies on a microscopic scale. They study the interaction among pedestrian systems, spatial structure, facility distribution, and human activities in the underground space of metro stations (15, 46, 47).

Most previous studies that have explored the linkage mechanisms of the built environment in MSAs and vibrancy are based on 5Ds (18, 19). However, the mechanisms by which the refined built environment of MSAs affects vibrancy are unclear. For example, the current residential and employment density data of the MSA are mainly derived from government census data (street level, TAZ) (49). However, the range of TAZ is much larger than that of MSA. It can lead to coarse measures of population density indicators and biased estimations. Moreover, the influence of three-dimensional built environment visual perception indicators such as green view index and sky rate on vibrancy remains to be explored. Therefore, it is necessary to extract and quantify more refined built environment elements in MSAs and analyze their relationship with vibrancy.

## Study area and data

### Study area

Chengdu, the capital of Sichuan Province, China, located in the Sichuan Basin, is an important central city in the western region. Chengdu is also an important national high-tech industrial base and integrated transportation hub. From 2010 to 2021, Chengdu's total population increased from 14 to 21 million, and the urbanization rate increased from 65% to 77%. Under the high population growth and urbanization process, Chengdu has become one of the cities in the world with the fastest urban rail transit construction. From the first line (Line 1) in Chengdu officially opened in September 2010 to the latest “five lines” of Chengdu Metro launched in December 2020, Chengdu metro soon became the fourth longest railway network in China in operation (only after Shanghai, Beijing, and Guangzhou). However, in the era of rapid population, economic development, and rapid urban rail transit construction, Chengdu's MSA construction and TOD vibrancy creation are significantly behind the speed of rail construction. In addition, since 2021, Chengdu has enacted urban development strategies, including the “*metropolitan area on the rail*” and “*TOD city*”, TOD has become an important urban development concept in Chengdu. Therefore, Chengdu- a TOD-informed megacity in China-is chosen as a case study.

**Figure 1:**
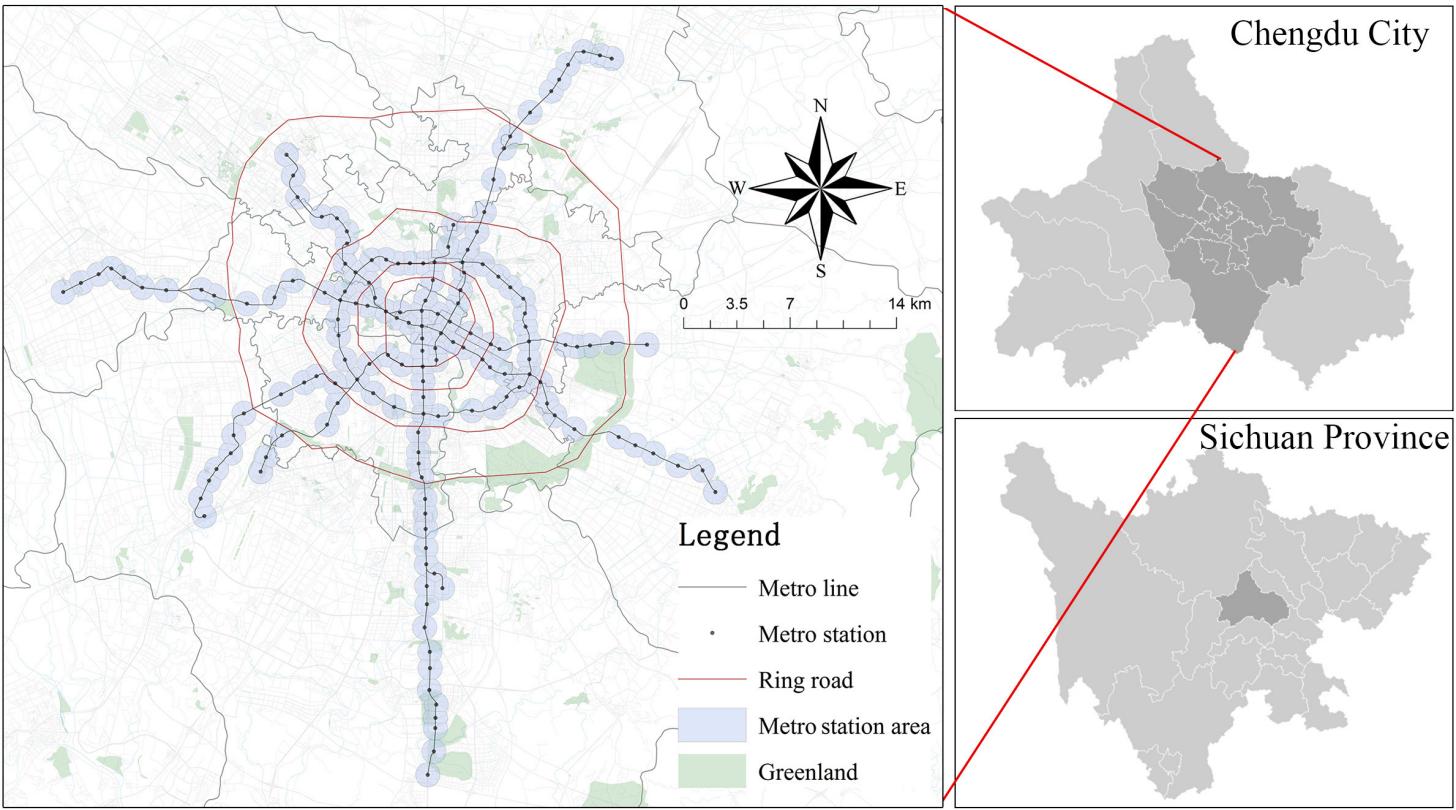
The study area and metro station area.

As of November 2019, there are six lines with a total of 156 stations (Figure 1). The rail lines in the study area cover a total of 10 districts in Chengdu, including five districts in the central area (Chenghua, Qingyang, Jinniu, Jinjiang, and Wuhou) and five districts in the periphery (Xindu, Pidu, Shuangliu, Wenjiang, and Longquanyi). To solve the buffer overlay problem of MSAs, our study on MSAs follows the work of Li et al. (50), and we use the 800 m buffer zone of stations as well as the Tyson polygon to determine the range of MSAs.

### Data sources

Considering that the COVID-19 epidemic broke out in December 2019, the mobile signaling data used in this study was dated November 2019.The mobile phone signaling data are obtained from DASS platform of SmartSteps, and we handle these data through Python and GIS. With the rapid spread of mobile communication and the Internet, individual/group mobile user activities generate continuous and abundant spatial position information. All the information forms the Mobile Positioning Big Data (MPBD), which records individual travel chains as well as massive crowd activity trajectories. Mobile phones have become one of the most important tools in residents' daily lives, and mobile communication networks in major cities have basically achieved full coverage (41). In 2019, the total number of mobile communication users of China's three major operators (China Mobile, China Telecom, and China Unicom) reached 1.6 billion. Given that mobile phone signaling data can reflect the spatio-temporal gathering patterns of the crowd (e.g., the distribution of working and living populations, user activity patterns), it is an important data basis for analyzing urban human activities. Compared with social network data such as Weibo and Facebook, mobile phone signaling data have larger data volume, more general coverage, and higher point density. It can accurately reflect the population attributes of users (age, gender, etc.). In this study, in order to ensure privacy and security, the mobile phone signaling data we obtain do not contain personal information. Moreover, our study divides the population structure into four levels based on the age classification standard of the World Health Organization (WHO) and the relevant regulations (e.g., *Chinese Law on the Protection of the Rights and Interests of the Elderly*). The classification in this work is as follows: (1) 12–18 years old (teenagers), (2) 19–39 years old (young adults), (3) 40–59 years old (middle-aged adults), and (4) 60+ (older adults).

**Figure 2:**
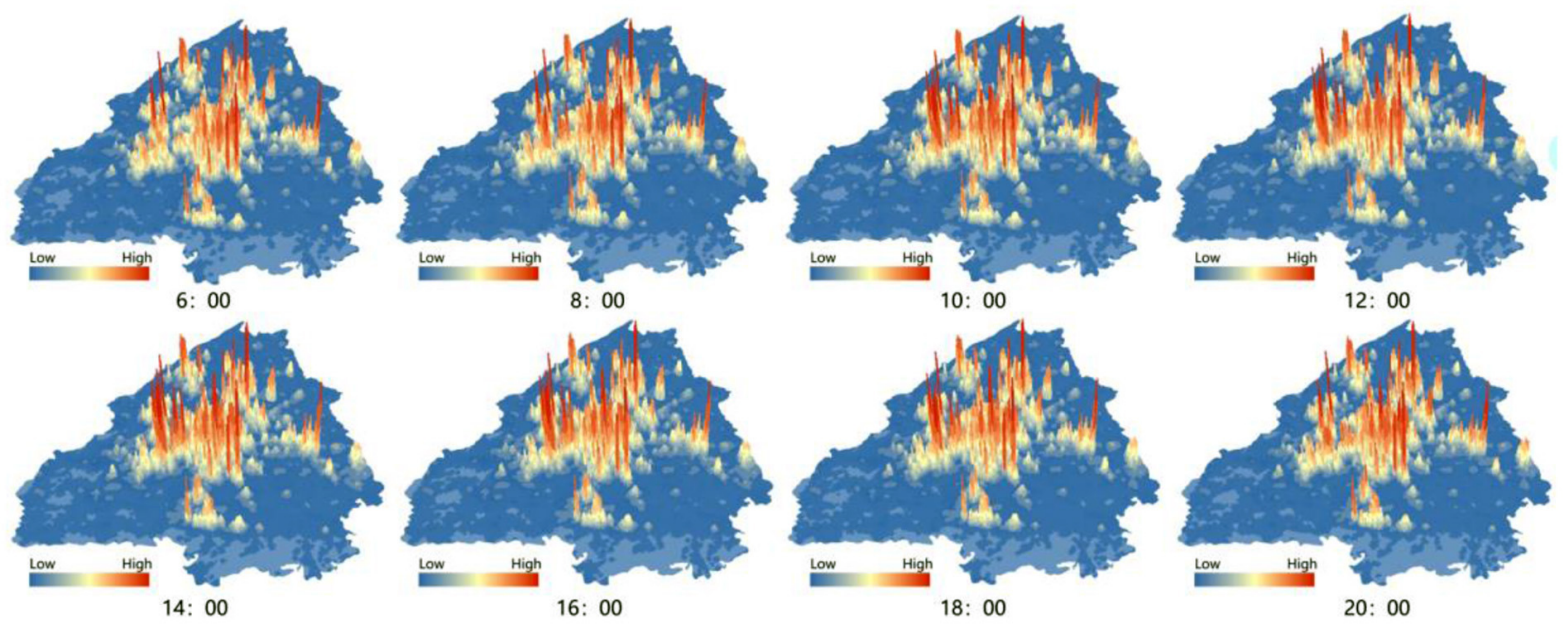
The heat map of population distribution in Chengdu.

According to the base station coverage of cell phones and the scale of neighborhoods (based on literature studies and the Chinese Ministry of Housing and Urban-Rural Development's 2022 “Annual report on road network density and traffic operation in major Chinese cities,” the average street density in Chengdu has reached 8.4 km/km^2^ and the street spacing is 250 m), in order to present street-level activity travel trajectories, we divided the study area into a grid of 250^*^250 m (Figure 2).

**Figure 3:**
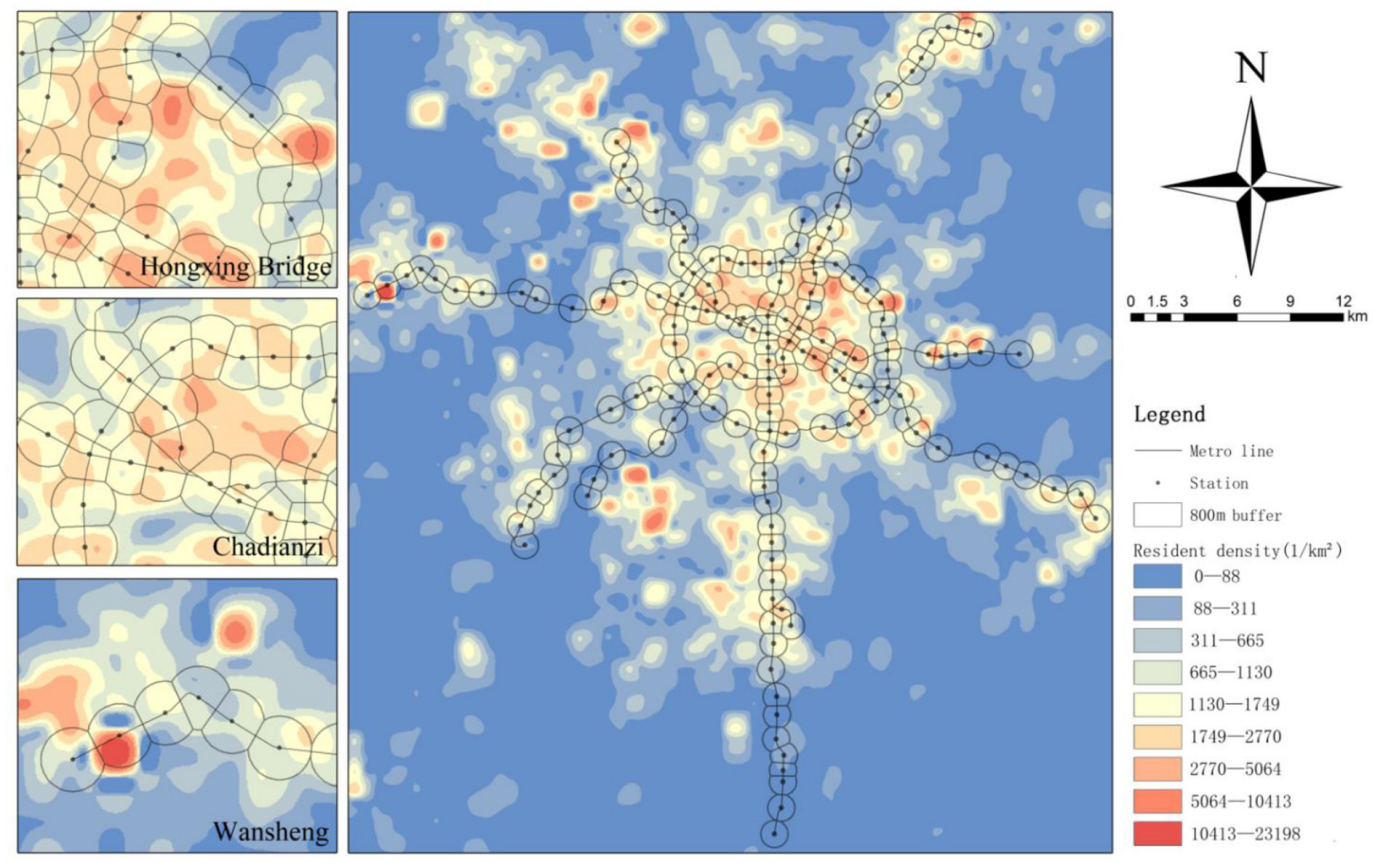
The identification and distribution of residential density in Chengdu based on mobile phone data.

Firstly, based on the method of Xiao et al. (51), we used mobile phone signaling data to calculate the number of residential and employment populations in each grid (Figure 3). Compared with the traditional census data, these data carved by mobile phone signaling are more refined. Secondly, we recorded the change in the population data within each grid, 24 h per day from November 11 to 17. It is worth noting that almost all previous studies based on transient tweets, hotspots, and other data, which recorded instantaneous population and integrated this into vibrancy. However, instantaneous population distributions are highly mobile, and many of the flows only pass through the area. For example, a person who drives through an MSA, even if he/she doesn't work or consume in the MSA, is still recorded as a vibrant individual. Thus, to extract the real vibrant users who generate consumption and staying time in the MSAs, we counted users who stayed in the MSA for more than 30 min and defined them as people with vibrancy. Finally, we aggregated the number of people with vibrancy in each grid to the MSA and calculated the average number of people with vibrancy per hour for each age group, recording the average TOD vibrancy index (VI) for each age group.

In recent years, POI data become an important representation of the distribution of facilities in the built environment. POI data record a series of information such as facility classification, name, and address. Compared with traditional land-use data, POI data (https://www.amap.com/) can fully reflect the characteristics of functional urban facilities' layout. The POI data we used in this study includes shopping facilities (Supermarkets, convenient stores, groceries, and so on), park facilities (plazas, parks, scenic areas, and ancillary facilities). Then we calculate the density of shopping facilities and park facilities. The CBD of Chengdu is Tianfu Square, so we calculate the distance to Tianfu Square of each metro station as the distance to CBD. The above indicators serve as destination accessibility of MSA-level built environment.

**Figure 4:**
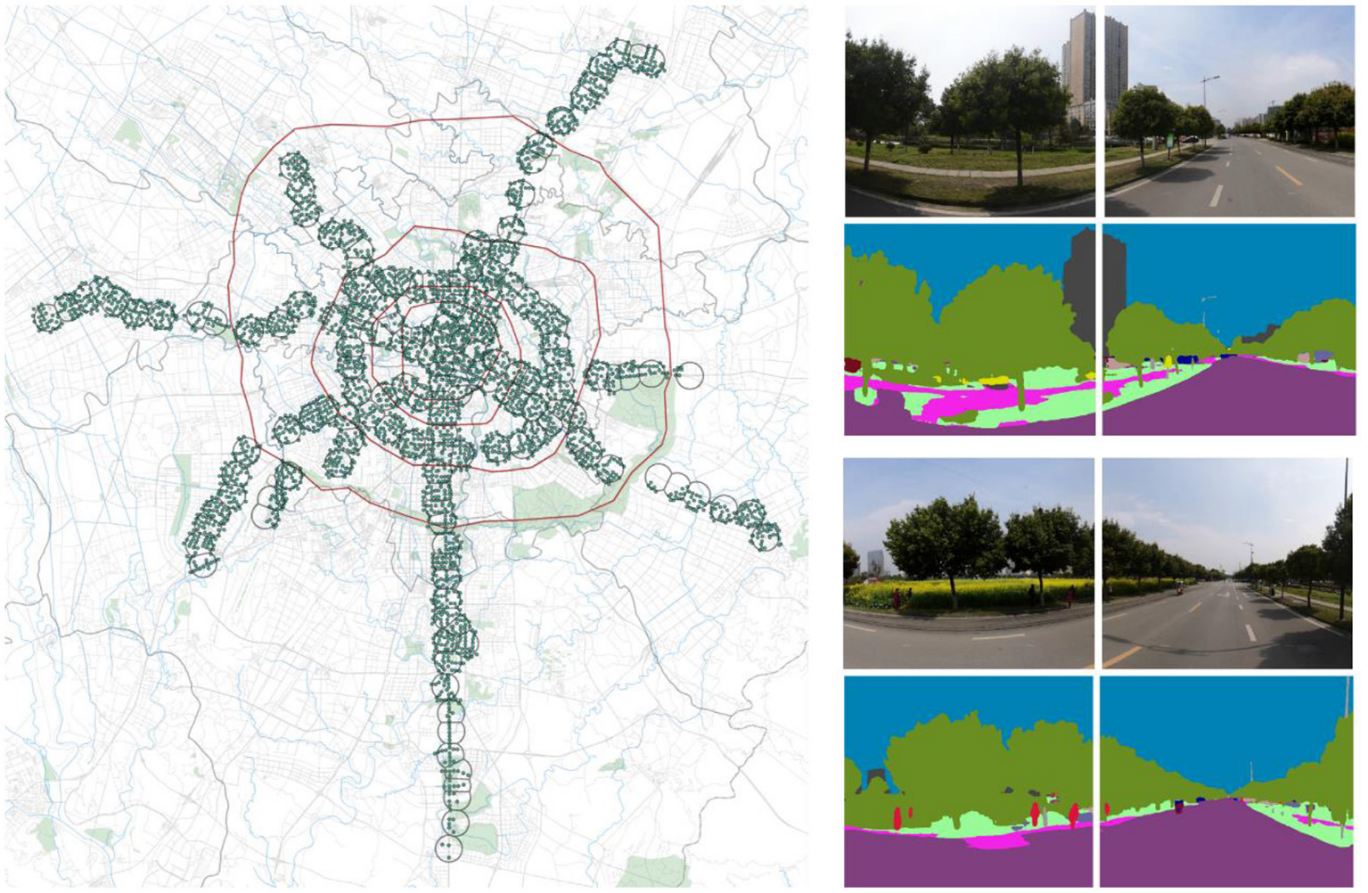
Street view point distribution in MSA and identification of green view rate.

In addition to POI data, the study also used urban spatial and economic data such as building footprint data (https://www.baidu.com/), road network data (https://www.openstreetmap.org/), housing prices (https://cd.lianjia.com), and bus routes and stops in Chengdu. Using These data, we calculated the FAR, road network integration index, average housing price, and bus stop density of MSA. We also calculated the land-use mix of MSA based on the POI data of Chengdu. Finally, we obtained the street view map of each MSA sampling point by calling the interface of Baidu API (Figure 4). Moreover, we calculated the street green view index of MSA by using a semantic image recognition tool. The green view index is calculated by


(1) $$VGI_{i}=\frac{\sum_{i=1}^{4}GreeneryPixels_{i}}{\sum_{i=1}^{4}Total\;Pixels_{i}}$$

where *VGI* is the green view rate and takes values in the range [0, 1), *GreeneryPixels*_*i*_ is the area of green pixel points of the *i*-th position in the street view map, and *TotalPixels*_*i*_ is the area of the observed street view image of the *i*-th position.

### Variables

The VI of TOD in this study is used as the dependent variable. Based on the 5Ds principle, and previous research (12, 17, 37, 42, 50), we selected built environment independent variables that may affect VI. The independent variables are as follow: density (residential density, employment density, and FAR), design (green view index and road network integration index), diversity (land-use mix), destination accessibility (distance to CBD, park density, and shopping facility density), and distance to transit (bus stop density). In addition, considering the economic attributes of MSA, we added the indicator of housing price as an independent variable. The descriptive statistics of the independent variables are shown in Table 1.

**Table 1:** Descriptive statistics of independent variables.

**Variables**		**SD**	**Mean**	**Min**	**Max**
Density	Residential density (10^4^/km^2^)	2.87	4.00	0.10	12.83
	Employment density (10^4^/km^2^)	4.67	3.51	0.11	29.75
	FAR (floor area ratio)	0.61	1.22	0.03	2.93
Diversity	Land-use mix	0.08	0.76	0.27	0.89
Design	Road network integration index	0.19	0.64	0.08	1.23
	Green view index (%)	0.05	0.19	0.05	0.32
Distance to transit	Bus stop density (1/km^2^)	4.74	8.51	0.57	27.36
Destination accessibility	Distance to CBD (km)	6.79	9.52	0.00	28.70
	Park density (1/km^2^)	5.50	3.08	0.00	39.13
	Shopping facility density (1/km^2^)	491.56	335.32	0.00	4,258.33
Economic attribute	Housing price (10^4^ yuan/m^2^)	0.49	1.56	0.38	3.54

### Methodology

**Figure 5:**
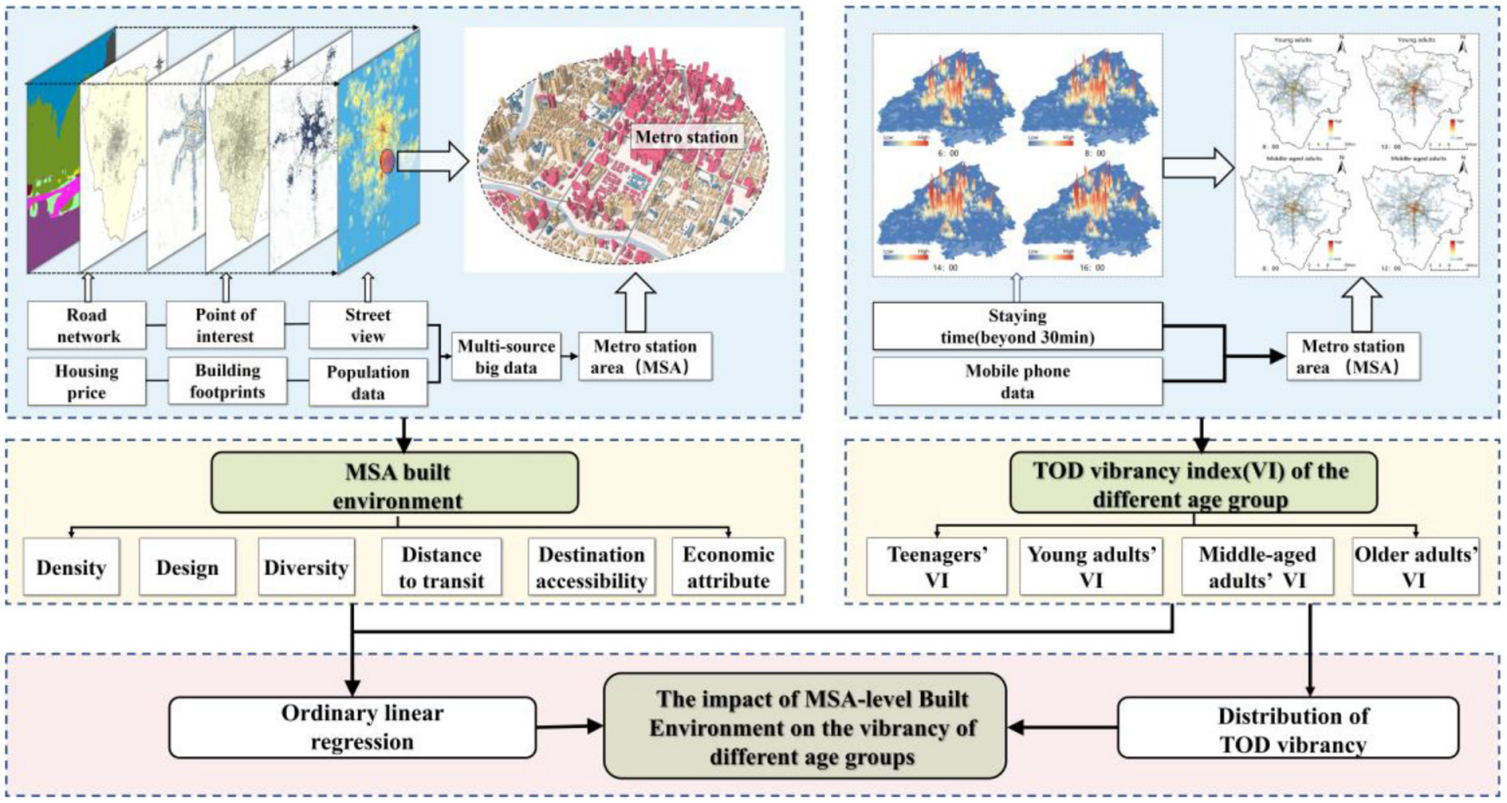
Methodological framework.

Our study explains the intergenerational differences in the impact of MSA built environment on VI employing a multiple linear regression model which illustrate the relationship between the independent and dependent variables (33), and the model is calculated by


(2) $$\begin{array}{l} \log y=\beta_{0}+\beta X+\varepsilon \end{array}$$

where *y* is the VI, *X* is a vector representing the independent variables, β_0_ is a constant, and β is the vector of estimated coefficients,ε is the residual. To investigate the intergenerational differences in the impact of the MSA-level built environment on VI, we employed five multiple linear regression model. Model 1 is a model of the impact on VI of all age groups, and Models 2–5 are models of the impact on the vibrancy of teenagers, young adults, middle-aged adults, and older adults, respectively. Separate models were constructed to explain the relationship between the MSA-level built environment and each age group's activities. The research framework of this paper is shown in Figure 5.

## Results

### Spatial distribution of all age group VI (Vibrancy Index) in MSA

**Figure 6:**
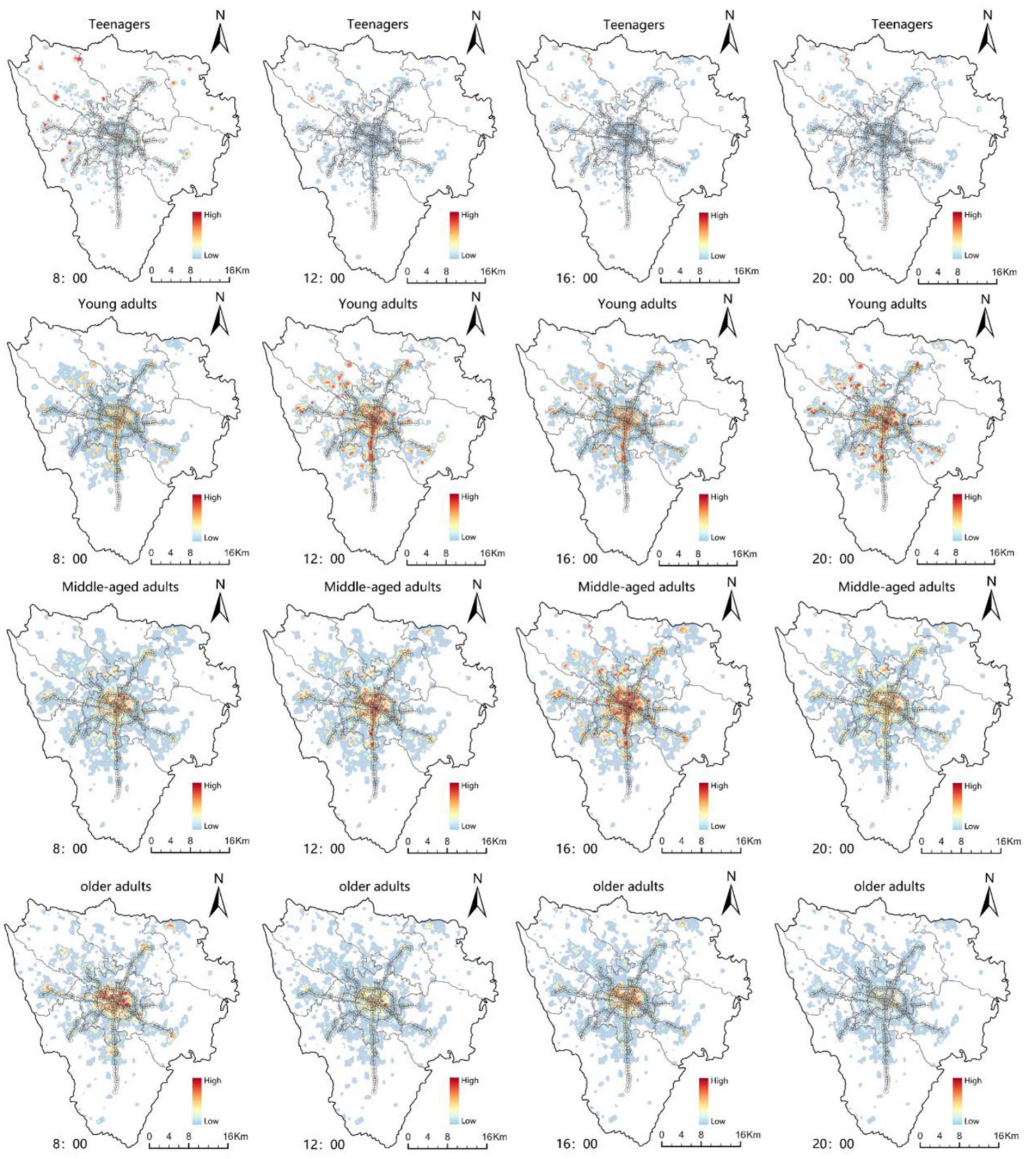
The distribution of TOD vibrancy for each age group (teenagers, young adults, middle-aged adults, and older adults).

(1) For teenagers (Figure 6), the MSAs in the central districts within the 3^rd^ Ring Road of the city are not the main concentration areas. Rather, it is the vocational-technical schools in the outer areas of the city that are the hotspots of teenagers' VI. We believe that two reasons may contribute to the spatio-temporal different characteristics of the teenagers' activities hotspots. Firstly, cell phones are banned in most Chinese primary, middle and high schools during school hours, while they are not in vocational-technical schools. Therefore, the recorded mobile phone signaling data of students in vocational-technical schools are likely to be more active. Secondly, due to vocational-technical schools' large student population and floor area, they are generally set up around MSAs in suburban areas. Thus, teenagers' VI is higher in the outer areas of the city. (2) For young adults, the Gaoxin South, Tianfu Square, and the Gaoxin West are the three major concentration areas. Tianfu 3^rd^ Street Station, Tianfu 5^th^ Street Station, Financial City Station, Chunxi Road Station, and Century City Station, which are located in these areas, combine the highest employment density in the city. Secondly, the central towns of the satellite cities are also secondary hotspots of young adults' VI. The end stations of several metro lines set near these towns (Xipu Station, Longquanyi Station, Science City Station, Wansheng Station, etc.) are highly attractive to the surrounding employment population, driving the employment of youth in rural. (3) There is some similarity in the distribution of VI between middle-aged adults and young adults, while middle-aged adults' VI is significantly higher in the city center than in several other age groups. The result may be strongly related to housing affordability and the consumption level of the middle-aged adults. (4) The older adults' VI is mainly located in the core metro stations in the city's central areas. Given that the MSAs within the 3^rd^ Ring Road have a huge number of old neighborhoods and high-quality medical services (e. g. Huaxi Hospital, Sichuan Provincial People's Hospital, etc.), the distribution of older adults' activities reveals a pattern of high in the center and low in the periphery.

### Results of the multiple linear regression model

**Figure 7:**
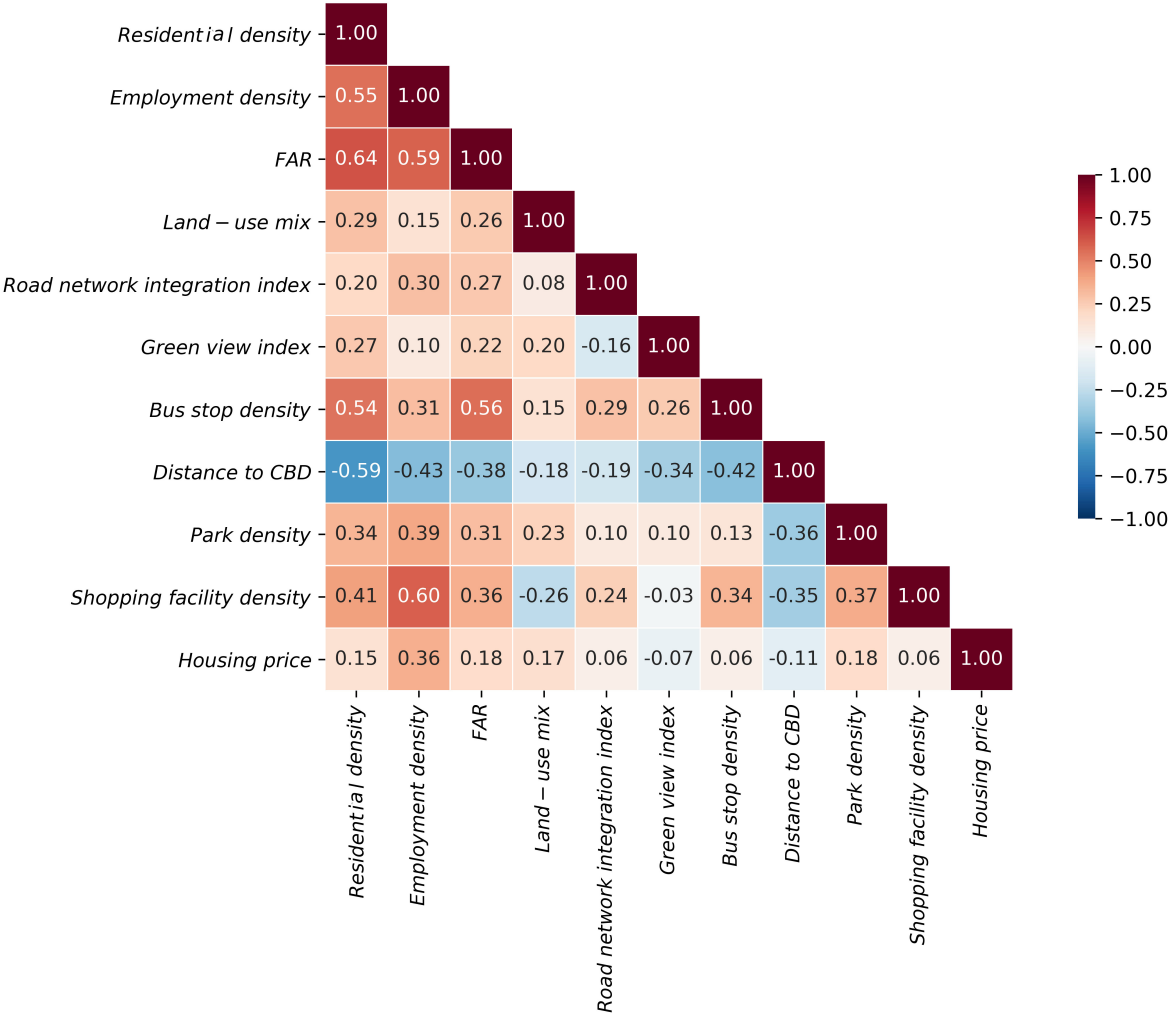
Pair-wise correlation analysis result.

Prior to the regression model calculations, we analyze the correlations between the built environment indicators through Stata (Figure 7). The results show that the correlation coefficients between indicators were all below 0.7, implying that there is no multicollinearity between these indicators. The results of the regression model reveal that the *R*^2^ of the overall model is 0.755 and the *R*^2^ of each age group model is 0.587 (teenagers), 0.719 (young adults), 0.758 (middle-aged adults), and 0.799 (older adults), respectively (Table 2). Plus, the model is relatively good with a high goodness-of-fit. In terms of VI for all ages, indicators including residential density, employment density, FAR, bus stop density, and green view index are significantly and positively correlated. The results of Zhang et al. (3) and Wu et al. (37) also support these findings. Meanwhile, indicators such as distance to the CBD and housing prices are significantly and negatively related to the VI. Surprisingly, land-use mix is not remarkably related to VI, which is different from the study by Yue et al. (40).

**Table 2:** Results of the multiple linear regression model.

**Variables**	**Model 1** **(overall)**		**Model 2** **(teenagers)**		**Model 3** **(young adults)**		**Model 4** **(middle-aged adults)**		**Model 5** **(older adults)**	
	**Coefficient**	**SE**	**Coefficient**	**SE**	**Coefficient**	**SE**	**Coefficient**	**SE**	**Coefficient**	**SE**
CONSTANT	9.916^***^	0.47	4.499^***^	1.066	9.342^***^	0.521	8.724^***^	0.426	7.756^***^	0.481
Residential density	0.123^***^	0.019	0.143^***^	0.044	0.137^***^	0.022	0.083^***^	0.018	0.121^***^	0.02
Employment density	0.044^***^	0.013	0.033	0.029	0.065^***^	0.014	0.023^**^	0.011	−0.007	0.013
Distance to CBD	−0.017^**^	0.007	−0.039^**^	0.016	−0.011	0.008	−0.024^***^	0.006	−0.034^***^	0.007
FAR	0.187^**^	0.089	0.147	0.201	0.121	0.098	0.241^***^	0.08	0.332^***^	0.091
Housing price	−0.466^***^	0.079	−1.073^***^	0.18	−0.48^***^	0.088	−0.393^***^	0.072	−0.495^***^	0.081
Bus stop density	0.031^***^	0.01	0.045^*^	0.023	0.028^**^	0.011	0.034^***^	0.009	0.040^***^	0.01
Shopping facility density	−0.001	0.001	0	0.003	−0.002^*^	0.001	0	0.001	0.001	0.001
Land-use mix	−0.163	0.534	−0.478	1.213	−0.112	0.593	−0.494	0.484	0.249	0.547
Road network integration index	0.111	0.212	0.926^*^	0.482	0.149	0.236	0.15	0.192	0.046	0.217
Park density	0.006	0.008	0.011	0.018	0.004	0.009	0.008	0.007	0.012	0.008
Green view index	1.459^*^	0.85	5.563^***^	1.931	1.098	0.944	1.592^**^	0.771	2.301^***^	0.871
**Performance statistics**										
*F*-statistic	40.416		18.588		33.452		40.930		52.184	
*R*-squared	0.755		0.587		0.719		0.758		0.799	
Number of observations	156		156		156		156		156	
Akaike crit. (AIC)	195.696		451.599		228.268		165.020		203.085	

*** p < 0.01.

** p < 0.05.

* p < 0.1.

The model results of each age group show that residential density (*p*-value < 0.01) and bus stop density contribute significantly to VI for teenagers, young adults, middle-aged adults, and older adults. There is little difference in the size of the coefficients. For example, bus stop density is significantly and positively correlated with VI for all ages, with coefficients ranging from 0.028 to 0.045. This suggests that residential density and transportation accessibility have a dominant effect on TOD vibrancy of all age groups. However, distance to CBD, FAR, housing price, employment density, green view index, shopping facility density, and road network integration index varies significantly on VI across all age groups. This finding suggests that there are considerably intergenerational differences in the impact of spatial construction intensity, street space design, and economic attributes of MSAs on TOD vibrancy.

## Discussion

### Density

Convincingly, residential density is significantly and positively correlated with VI for all ages. This is consistent with the conclusion of Yue et al. (40) and Wu et al. (52). Currently, most schools and companies in China institute an 8-h working model. For most people, apart from travel activities such as working, going to school, and hanging out, the remaining time of daily activities are spent around their living places, including consumption and leisure. Thus, compared to development density and employment density, residential density is the core of human activities that guarantee the basic source of TOD vibrancy. As we expected, employment density is not correlated with the older adults' and the teenagers' VI but is positively correlated with young adults' and middle-aged adults' VI. Moreover, the influence coefficient of young adults' VI is 0.065 (*p*-value < 0.01), which is higher than that of middle-aged adults' VI (0.023, *p*-value < 0.05). Consistent with the employment density distribution in Shanghai, Tokyo, Seoul, and Hong Kong (7, 53–55), Chengdu's MSA is the area with the highest employment density in the city. And both the young adults and the middle-aged adults are the main population currently employed. In this regard, it is reasonable to assume that VI for these two age groups are positively correlated with MSA employment density. In addition, the working model of “996” (work from 9 am to 9 pm per day, 6 days per week) is now common in companies (51). Compared to the middle-aged adults, “generation oppression” at the workplace (56) usually extends more overtime work for young adults. So, the population and gathering time of young adults are more than other age groups in MSAs with higher employment density. To be sure, this phenomenon is also common in other East Asian countries such as Korea and Japan (56). Then, as for the impact of FAR on VI, there is also a notable intergenerational difference. And the impact of FAR on VI continued to increase with age. We believe that this may be strongly related to the urban spatial and population structure of Chengdu: Firstly, TOD development in the central areas of Chengdu is earlier, resulting in a considerably higher MSA development intensity (FAR) than that in the peripheral areas. Secondly according to data from the “*The seventh census of China*,” old neighborhoods around city center are now the main settlement areas for the older adults. Therefore, MSAs with a high FAR in the central part of the city are more active for older adults.

### Diversity

According to the New Urbanism and Smart Growth, the land-use mix model is important to enhance urban vibrancy (3, 57, 58). Meanwhile, it is surprising that land-use mix is not associated with VI in either the overall model or the models for each age group, which is remarkably different from previous studies. For instance, some empirical studies in cities such as Shenzhen and Seoul show that land-use mix and spatial interaction diversity have a significant contribution to urban vibrancy (40, 59, 60). The reason may be as follow: People are able to accomplish their travel purposes in a relatively small range in TOD with a high land-use mix, which may restrict the mobility and redistribution of people. The study by De Nadai, Staiano (36) based on cities in Italy also finds that there is no significant relationship between land diversity and urban vibrancy. Thus, the mixed development model advocated by New Urbanism should base on the high quality of space.

### Destination accessibility

Distance to CBD is significantly and negatively related to teenagers', middle-aged adults', and older adults' activities, and the coefficients vary little. The geographic decay function for urban human activities is also evidenced in studies by Tu et al. (34) and Yang et al. (61). It is worth noting that young adults' activities are less influenced by distance, and there is no remarkable relationship. We believe that this may be related to the polycentric development pattern of Chengdu and the spatial movement patterns of the young adults. Firstly, the employment centers in Chengdu are located in the MSAs of new urban areas such as the Gaoxin South, the Gaoxin West, and Tianfu New District, while the commercial centers (consumption centers) are located in the MSAs of old urban areas such as Tianfu Square and Chunxi Road Station. Secondly, young adults are more active and mobile compared to other age groups. As the main population of employment and consumption in the city, their activities pattern presents a homogeneous distribution throughout the day. This results in the fact that the young adults are less influenced by the geographic decay function of “center-periphery” distance in the city.

### Design

The green view index is significantly and positively correlated with VI for teenagers, middle-aged adults, and older adults. With coefficients of 5.56, 1.59, and 2.30, respectively. The existing literature shows that the green view index is an important impetus to enrich human activities, as greenery can improve the pedestrian walking experience by increasing shade and reducing stress (62). Older adults have a more flexible activity schedule and are more focused on physical health and physical activities. Thus, MSAs with a high green view rate are attractive to senior citizens. As for the teenagers, green space makes up a large part of campus spaces in China, so teenagers' VI is also significantly and positively correlated with MSA green view index rates. Meanwhile, the effect of the green view index on the young adults' VI was not remarkable. This is a very interesting result, and we think the possible reasons are as follows: (1) Due to economic pressure, the young adult group is more concerned with the economic benefits of TOD rather than spatial quality. (2) Given that MSAs with high employment density and high development intensity, which highly attract young adults, are generally new urban areas. The green view rate of street space in new areas is relatively lower because of the wider streets and the shorter planting time of greenery. Therefore, under the combined influence of group activity preferences and MSA place-making differences, the use of green space in TOD is “spatially inequity” for young adults.

The road network integration index is an important spatial design indicator of TOD street networks. Hillier et al. (63) argued that healthy urban spaces attract pedestrian flows and further attract specific functional spaces to grow in density, which in turn leads to a high consistency in the scale of pedestrian flows and the structure of the street network. So, integration is the core of functions and pedestrian flows aggregation. However, the model results show that the road network integration index is only positively related to teenagers' VI, but not significantly related to VI for other age groups. We believe that this may be related to the spatial structure of road networks in Chengdu. The road network structure within the 3^rd^ Ring Road in Chengdu has retained small-scale neighborhoods and complex road networks, resulting in low road network integration index of MSAs. By contrast, the new areas outside the 3^rd^ Ring Road have grid-like and highly integrated road network structures. The road network integration index of MSA in the periphery area of the city is higher than that in the central of the city. The distribution of VI for different age groups shows a spatial pattern that is high in the center and low in the periphery, except that the distribution of teenagers' VI was on the contrary. In this regard, it is understandable that the other age group activities do not correlate with the road network integration index of the MSA.

### Distance to transit

The bus stop density is highly consistent for all age group models, with little intergenerational differences. Compared to the metro system, bus transit has much greater coverage and is important for connecting other areas of the city to the MSA. Besides, transit accessibility has been noted in many studies to increase metro ridership (64), which is the most important source of human activities in MSAs. Therefore, MSAs with higher bus stop density have a greater capability to radiate and attract people from surrounding areas.

### Housing price

Housing prices are negatively correlated with VI for all age groups, suggesting that high housing prices in MSAs may inhibit the TOD vibrancy. We think that this may be related to the “spatial squeezing effect” of high housing costs. High housing prices in TOD can “squeeze” out non-housing affordable people from MSAs and urban centers (65). Then, under the constraints of market choice and payment capability, low- and middle-income people often have to give up accessibility for relatively affordable houses. This social phenomenon results in a spatial mismatch between work and housing and a significant time spent on commuting. So, it can be explained that “spatial squeeze” and “jobs-housing imbalance” generates negative externalities, which in turn inhibit the time and density of people gathering in MSAs.

Previous studies have shown that urban rail transit has a positive impact on housing prices (66). On one hand, according to classical land economics and ground rent theory, urban metro construction enhances the spatial accessibility of stations and areas along the rail lines, rising land and housing prices (67). On the other hand, TOD-related facilities are easily capitalized into neighboring property values. Because people are willing to pay a higher price for convenient and efficient access to public services and infrastructure. Some scholars refer to this effect as “transit capitalization effects” or the “value capture” of rail transport. Similarly, in countries such as India, Korea, and China, where rapid urbanization is taking place, TODs are more used as a tool for real estate development to promote urban development and land taxation. In short, this process of TOD-based real estate appreciation is accompanied by a continuous division of classes and generations, coming into “Transit-Induced Gentrification” (68). Chava et al. (69) have pointed out that the gentrification of MSAs has a significant relationship with people's usage frequency of rail transport.

Surprisingly, MSA housing prices have the most impact on the suppression of teenagers' VI. We suggest that this may be closely associated with the “school district housing” and “squeeze of land value.” For one reason, China's policy of the School District System (70) results in the housing price rising in MSAs with better educational resources in central areas. This leads to a large number of teenagers moving with their parents to study in city peripherals. For another reason, the abundant added value of land in MSAs with high housing prices will lead to a tendency to develop commercial and residential facilities, which will squeeze out space for larger public infrastructure such as education and sports. This cycle further exacerbates the suppression on teenagers' VI in MSAs with high housing prices.

## Conclusions

Transit-oriented development has been touted as an important integrated transport and urban planning concept for enhancing urban vibrancy and realizing sustainable urban development. In this study, we use cellular signaling data to quantify the spatio-temporal distribution of the MSA-level VI among different age groups. Meanwhile, multi-sourced spatial data such as streetscape, parcel-level land use, and POI data are adopted to measure the MSA-level refined built environment. Based on these data, we apply multiple linear regressions to scrutinize the impact of the MSA-level built environment on the VI and its intergenerational differences.

The findings of the study are conducive to understand the mechanisms of how the MSA-level built environment affects urban vibrancy and to inform urban planners on how to create vibrant TOD communities. Specifically, the visualization results show that there are evident intergenerational differences in the spatial distribution of the MSA-level VI. The MSA in new urban areas, in which plenty of firms are concentrated, accommodates a large number of activities of young and middle-aged adults. In contrast, the hotspots of the MSA-level activities of older adults are mainly located in the well-developed city center with premium accessibility to public service facilities. Regarding the impact of the MSA-level built environment factors, the results of regression analysis indicate that (1) Residential and bus stop density are positively associated with the VI. Furthermore, most of the correlation coefficients are similar among different age groups. (2) Distance to CBD is negatively associated with the VI of teenagers, middle-aged, and older adults, while it is unrelated to the VI of young adults. (3) Employment density is positively associated with the VI of young and middle-aged adults but not significantly associated with those of teenagers and older adults. (4) The correlations between the FAR and the VI are positive for all age groups. As age increases, the significance of such correlations becomes more pronounced. (5) Green view index shows a more significant positive correlation with the VI of teenagers and older adults as compared to those of young and middle-aged adults. (6) Significant negative correlations exist between housing price and VI of different age groups. Teenagers are influenced the most by such spatial-squeezing effects, i.e., transit-induced gentrification.

Based on these findings, we propose the following planning recommendations for enhancing TOD vibrancy. First, the planning and design of the TOD living circle need to take the intergenerational differences in human activities into consideration. For instance, TOD communities with a high fraction of older adults are supposed to provide sufficient elderly-friendly facilities and improve the streetscape design, thereby facilitating the mobility and activity of the older adults in the MSA. Second, to address the adverse effects of “Transit-Induced Gentrification” on urban vibrancy, we call for efforts to develop and improve the multi-level housing support system (e.g., government-subsidized and affordable rental housing) in the MSA to achieve a systematic spatial match for the “housing-transportation” system. This will help reduce the “Spatial Deprivation” suffered by low-and middle-income residents due to high housing prices (71), thus contributing to the development of equitable, accessible, and vibrant TOD communities. Finally, to accommodate the daily activities of young adults, urban planners need to improve the quality of public space in the high employment density MSA by providing adequate leisure facilities like small community parks.

However, there are limitations in this study. First, Meng and Xing (5) and Xiao et al. (42) considered urban vibrancy as an aggregative index, so they aggregated data from social media, smartcards, and POIs to form a comprehensive urban vibrancy index. Meanwhile, our study uses mobile phone signaling data, which mainly responds to the density and duration of people aggregation. Thus, the calculation of the vibrancy index for each age group based on this dataset may lead to an incomplete characterization of urban vibrancy. Secondly, although we use Tyson polygons to reduce the effect of MSA spatial proximity on the model results, it is still difficult to solve the problem of spatial heterogeneity. In this regard, the next study should employ a geographically weighted regression model (GWR) or a geographically temporally weighted regression model (GTWR) to reduce the effect of spatio-temporal nonstationary on the model results.

## Data availability statement

The original contributions presented in the study are included in the article/supplementary material, further inquiries can be directed to the corresponding authors.

## Author contributions

BY, XC, and FT: conceptualization. BY and XC: funding acquisition. BY: supervision and writing—original draft. XC and FT: methodology. RL and FT: formal analysis. XC, RL, FT, TY, and PL: writing—review and editing. PL, TY, and FT: validation. All authors contributed to the article and approved the submitted version.

## Funding

This study was supported by the Doctoral Innovation Fund Program of Southwest Jiaotong University (No. 2017310253) and the National Natural Science Foundation of China (No. U20A20330).

## Conflict of interest

The authors declare that the research was conducted in the absence of any commercial or financial relationships that could be construed as a potential conflict of interest.

## Publisher's note

All claims expressed in this article are solely those of the authors and do not necessarily represent those of their affiliated organizations, or those of the publisher, the editors and the reviewers. Any product that may be evaluated in this article, or claim that may be made by its manufacturer, is not guaranteed or endorsed by the publisher.
